# Baseline Serum Cystatin C Is a Potential Predictor for Acute Kidney Injury in Patients with Acute Pancreatitis

**DOI:** 10.1155/2018/8431219

**Published:** 2018-11-19

**Authors:** Xue Chai, Hou-Bao Huang, Gang Feng, Yu-Han Cao, Qing-Shui Cheng, Sheng-Hui Li, Chi-Yi He, Wei-Hua Lu, Ming-Ming Qin

**Affiliations:** ^1^Department of Urology, The First Affiliated Hospital of Wannan Medical College, Wuhu, 241001 Anhui, China; ^2^Clinical Laboratory, The First Affiliated Hospital of Wannan Medical College, Wuhu, 241001 Anhui, China; ^3^Department of Nephrology, The First Affiliated Hospital of Wannan Medical College, Wuhu, 241001 Anhui, China; ^4^Department of Gastroenterology, The First Affiliated Hospital of Wannan Medical College, Wuhu, 241001 Anhui, China; ^5^Department of Intensive Care Unit, The First Affiliated Hospital of Wannan Medical College, Wuhu, 241001 Anhui, China

## Abstract

**Aims:**

The study is aimed at studying the incidence of acute kidney injury (AKI) and exploring the potential predictor for AKI in patients with acute pancreatitis.

**Methods:**

A retrospective study adopting a stratified cohort sampling design was performed in a cohort of patients (*n* = 237) diagnosed with acute pancreatitis without any renal injury. The following information including age, gender, serum creatinine, serum urea nitrogen, serum uric acid, serum cystatin C, fasting serum glucose, serum amylase, serum lipase, serum choline esterase, total protein, albumin, globulin, total bilirubin, direct bilirubin, total bile acids, glutamic-pyruvic transaminase, glutamic-oxaloacetic transaminase, gamma glutamyl transpeptidase, and alkaline phosphatase were collected from each patient when they were diagnosed with acute pancreatitis. Student *t*-test was conducted to figure out the difference between patients with and without AKI. Univariate and multivariate logistic regression analyses were used for investigating the predictors for AKI in patients with acute pancreatitis.

**Results:**

18 (7.6%) patients in total had developed AKI among the study group. Compared with patients without AKI (1.01 ± 0.26 mg/L), the level of baseline serum cystatin C (CYS-C) was significantly higher in patients with AKI (3.64 ± 2.17 mg/L, *P* < 0.001). Baseline serum CYS-C (OR = 203.594, *P* < 0.001) was the independent and significant predictor for AKI in patients with acute pancreatitis. AKI in patients with acute pancreatitis could be identified with a sensitivity of 88.9% at specificity of 100% (AUC = 0.948, 95% CI 0.879–1.000) by baseline serum CYS-C (cut-off value = 1.865 mg/L).

**Conclusions:**

Baseline serum CYS-C shall be adopted to predict the potential risk of AKI in patients with acute pancreatitis.

## 1. Introduction

Acute pancreatitis is an inflammatory disease of the pancreas, the most common cause for acute hospitalization among gastrointestinal diseases. There are about 20 to 80 patients susceptible to the disease per 100,000 annually, demonstrating a high incidence [1, 2]. In Shanghai, China, acute pancreatitis increased from 30.5 per 100,000 in 2009 to 39.2 in 2014 (an annual increase of 5.1%), among which the severe acute pancreatitis (SAP) marks the highest increase (13.7% annually) [3]. The mortality is as high as 30%, despite progress in recognizing the pathogenesis and implementing intensive care in patients with moderately severe acute pancreatitis (MSAP) and SAP [4], characterized by a complex and incompletely understood pathophysiological mechanism [5, 6]. Complications of acute pancreatitis include systemic inflammatory response syndrome (SIRS), sepsis, acute respiratory distress syndrome (ARDS), and acute renal injury (AKI). The prognosis of patients with acute pancreatitis is largely determined by the presence of organ failure and infected pancreatic necrosis with associated mortality rates of 15%–30% [7].

AKI is defined as the abrupt (within 48 h) and sustained decline of renal function [8]. The mechanisms for AKI development remain poorly understood; however, ischemia reperfusion, sepsis, and kidney oxygenation were considered to be one of the main mechanisms for AKI [9]. AKI is a contributing factor in the development and progression of chronic kidney disease (CKD), despite the fact that mechanism underlying AKI-CKD transition remains largely unclear [10]. Currently, the risk, injury, failure, loss, and end stage (RIFLE) and the Kidney Disease Improving Global Outcomes (KDIGO) guideline are adopted to diagnose AKI. Both criteria are based on the increase in serum creatinine and the decrease in diuresis [11]. Unfortunately, the finite value of serum creatinine in the early phase of AKI and an increase in serum creatinine concentration usually occur in about 1–2 days upon kidney injury [12].

When acute pancreatitis leads to AKI, there is a 5- to 10-fold rise in mortality, amounting to 70% [13–15]. Therefore, the control and prevention of AKI can be a useful strategy in the prevention of the morbidity and mortality associated with acute pancreatitis. For several decades, lots of biomarkers that can predict AKI were introduced to promptly initiate aggressive kidney-sparing and life-saving treatment [16]. These biomarkers mainly include kidney injury molecule-1 (KIM-1) [17], neutrophil gelatinase-associated lipocalin (NGAL) [18], and serum cystatin C (CYS-C) [19].

However, few studies have focused on the early markers of predicting AKI in patients with acute pancreatitis. Under such circumstances, this study is aimed at investigating the incidence of AKI and exploring potential predictor for AKI in patients with acute pancreatitis.

## 2. Methods and Subjects

### 2.1. Study Subjects

A retrospective study by a stratified cohort sampling design was conducted among a cohort of patients (*n* = 237) diagnosed with acute pancreatitis without any renal injury. These data of patients with acute pancreatitis were obtained from the First Affiliated Hospital of Wannan Medical College between April 2016 and May 2018. Acute pancreatitis was diagnosed under the 2012 revision of the Atlanta classification. A patient was diagnosed upon the presence of at least two of the three following symptoms: consistent abdominal pain with acute pancreatitis (acute onset and persistent and severe and epigastric pain); serum lipase or amylase activity at least three times greater than the upper reference limit; and the characteristic findings of acute pancreatitis on contrast-enhanced computer tomography or magnetic resonance imaging or transabdominal ultrasonography [20]. The subjects with chronic pancreatitis, neoplasms, and chronic liver diseases such as cirrhosis or viral hepatitis, chronic kidney diseases such as nephritis, or renal failure were not eligible to the baseline recruitment.

According to the clinical evaluation of the severity of acute pancreatitis, the patients were categorized into the following 3 groups: with mild acute pancreatitis (MAP), MSAP, and SAP. The MAP group was composed of patients who did not show any organ failure or local complications. Patients with organ failure lasting less than 48 hours (transient organ failure), local complications (necrosis, acute necrotic collection, and walled-off pancreatic necrosis), and/or exacerbation of comorbidity were categorized into MSAP. Patients with persistent organ failure (lasting more than 48 hours) and ≥1 local complication were assigned to SAP [20].

**Figure 1:**
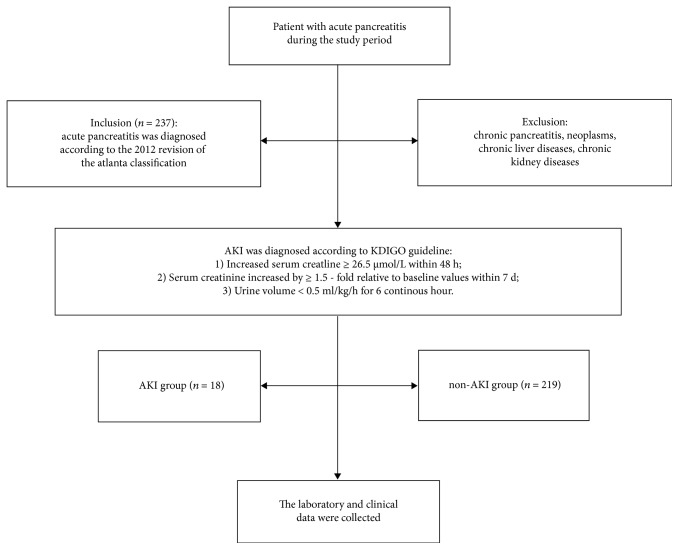
The flow diagram of patients.

All patients were classified into an AKI group (*n* = 18) and a non-AKI group (*n* = 219) according to dynamic changes in serum creatinine levels. AKI was diagnosed in accordance with KDIGO guideline [21], and the results were as follows: (1) increased serum creatinine ≥ 26.5 *μ*mol/L within 48 h; (2) serum creatinine increased by ≥ 1.5-fold relative to baseline values within 7 d; and (3) urine volume < 0.5 mL/kg/h for 6 continuous hours (Figure 1).

### 2.2. Method of Measurement

Serum creatinine (Cr), serum urea nitrogen (Bun), serum uric acid (UA), serum cystatin C (CYS-C), fasting serum glucose (FBG), serum amylase (AMY), serum lipase (LIP), serum choline esterase (CHE), total protein (TP), albumin (ALB), globulin (GLOB), total bilirubin (TBIL), direct bilirubin (DBIL), total bile acids (TBA), glutamic-pyruvic transaminase (ALT), glutamic-oxaloacetic transaminase (AST), gamma glutamyl transpeptidase (GGT), and alkaline phosphatase (ALP) were measured under the standard techniques by an automatic analyzer (Hitachi 7060; Hitachi High Technologies, Tokyo, Japan) upon the manufacturer's instructions.

### 2.3. Statistical Analysis

Continuous variables were normally distributed by Kolmogorov-Smirnov test and provided as mean ± SD. Categorical variables were expressed as percentages. Overall comparisons were performed with Student *t*-test. Differences in percentages of variables were determined by chi-squared test. Univariate logistic regression analysis and multivariate logistic regression model were conducted to predict AKI in patients with acute pancreatitis. ROC curve and the corresponding area under the curve (AUC) were calculated for testing the potential of serum CYS-C, Bun, Cr, and UA for AKI in patient with acute pancreatitis. All statistical analyses were performed under the SPSS 19.0 version (SPSS Inc., Chicago, IL, USA) and R version 3.3.2. A two-sided *P* value < 0.05 was considered statistically significant.

## 3. Results

A total of 237 patients with acute pancreatitis were collected in this study, including 108 females (45.57%) and 129 males (54.43%), and the mean age was 58.81 ± 15.46 years (range 20–85 years). The number of patients with MAP, MSAP, and SAP were 187, 38, and 12, respectively (78.90%, 16.03%, and 5.06%). There were 18 (7.6%) patients who had developed AKI according to the KDIGO guideline. The number of patients with stage I, II, and III were 9, 7, and 2, respectively (50.00%, 38.89%, and 11.11%). The male (10.9%) was higher than female (3.7%, *P* < 0.05).

Baseline characteristics of all subjects are summarized in Table 1. No difference was observed in age (*P* = 0.733), FBG (*P* = 0.422), AMY (*P* = 0.220), TP (*P* = 0.082), ALB (*P* = 0.057), GLOB (*P* = 0.286), TBIL (*P* = 0.473), DBIL (*P* = 0.225), TBA (*P* = 0.226), ALT (*P* = 0.876), AST (*P* = 0.432), GGT (*P* = 0.722), and ALP (*P* = 0.560) between patients with and without AKI. Compared with acute pancreatitis patients without AKI, acute pancreatitis patients with AKI had higher serum Bun, higher serum Cr, higher serum UA, higher serum CYS-C, lower serum LIP, and lower serum CHE (*P* < 0.05).

**Figure 2:**
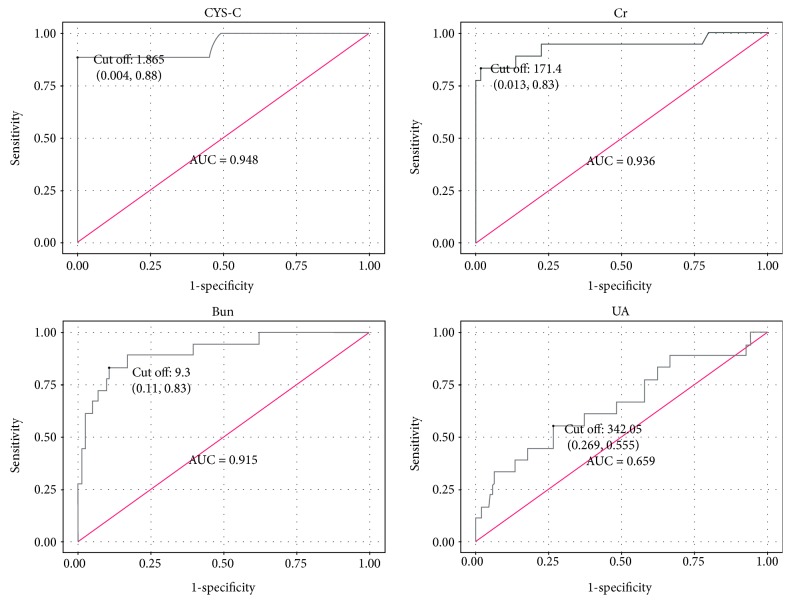
Receiver operating characteristic (ROC) curve of serum CYS-C, Bun, Cr, and UA for AKI in patient with acute pancreatitis.

By univariate logistic regression analysis, gender (OR = 0.316, *P* = 0.048), serum CHE (OR = 0.999, *P* < 0.001), TP (OR = 0.886, *P* = 0.003), ALB (OR = 0.865, *P* = 0.005), serum Bun (OR = 1.449, *P* < 0.001), serum Cr (OR = 1.044, *P* < 0.001), serum UA (OR = 1.006, *P* = 0.002), and serum CYS-C (OR = 284.266, *P* < 0.001) were the significant indicators for AKI in patients with acute pancreatitis (Table 2). According to multivariate logistic regression analysis, serum CYS-C (OR = 203.594, *P* < 0.001) was the independent and significant indicator for AKI in patients with acute pancreatitis (Table 3). As shown in Figure 2 and Table 4, AKI in patients with acute pancreatitis could be identified with a sensitivity of 88.9% at specificity of 100% (AUC = 0.948, 95% CI 0.879–1.000) by baseline serum CYS-C (cut-off value = 1.865 mg/L).

## 4. Discussion

AKI is a clinical syndrome defined as the abrupt (within 48 h) and sustained decline of renal function caused by a variety of factors including insufficient renal blood perfusion, renal toxic substances, inflammatory factors, and urinary tract obstruction. At present, there is still no specific treatment in clinical practice, and the mortality rate remains high. AKI, a complication of acute pancreatitis, has been reported with an incidence between 1% and 14%, while mortality with acute pancreatitis ranges from 0 to 30% [22]. However, among a total of 414 SAP patients in the intensive care units (ICU), 287 (69.3%) developed AKI during their stay [23]. In this study, 18 (7.6%) patients had developed AKI in line with KDIGO guideline, due to most of the patients were MAP and MSAP. There was a higher percentage of AKI in males (10.9%) than in females (3.7%). This result was consistent with previous reports [3], suggesting that male patients with acute pancreatitis were more apt to developing AKI.

Studies showed that more than a half of deaths among SAP patients occurred within the first week of acute pancreatitis [24]. AKI is the most common cause of mortality in SAP patients [11, 23]. Thus, early interventions of AKI are urgently needed for the patients in this critical stage. Although the pathogenesis of AKI in SAP is not fully elucidated, the effect of pancreatic amylase on the renal microcirculation, hypoxemia, or a toxic effect of excessively produced pancreatic phospholipase A2 on the proximal tubules may lead to AKI. These factors cause a decrease in renal perfusion and damage to the renal tubules and the increase in small molecule substance concentrations [12] consequently. Multiple new molecular markers have been found to predict AKI in patients at the early stage. Shaker et al. found that plasma fibroblast growth factor-23 (FGF-23) was a novel and highly predictive early biomarker for AKI after cardiac surgery [25]. Pang et al. found that urinary KIM-1 and urinary NGAL could efficiently discriminate patients with and without vancomycin-associated AKI earlier than serum Cr [26]. In addition, neonates with respiratory distress syndrome (RDS) are at higher risk for AKI. Serum CYS-C on day 3 of life can predict AKI earlier than serum Cr and eGFR [27]. However, the predictors of AKI in patients with acute pancreatitis are unclear.

In this study, liver function indexes (TP, ALB, GLOB, TBIL, DBIL, TBA, ALT, AST, GGT, and ALP) had no differences between acute pancreatitis patients with AKI and without AKI. These results suggested that liver function indexes might not be adopted to predict AKI in patients with acute pancreatitis. Furthermore, the high level of serum Bun, serum Cr, and serum UA were found in patients with AKI. These results suggested that AKI in patients with acute pancreatitis have a greater increase because of injury of renal function in these subjects. The low level of serum LIP and serum CHE were found in patients with AKI. Univariate logistic regression analysis demonstrated that these variables might be significant indicators for AKI in patients with acute pancreatitis.

Currently, serum Cr, serum Bun, and urine volume are used to evaluate kidney function. However, these indices are extremely limited for the early diagnosis of kidney injury [28] and are easily affected by some nonrenal factors [29]. Serum CYS-C is a cysteine proteinase enzyme inhibitor, and it has low molecular weight and does not bind to proteins. It is freely filtered by glomerulus [30]. Upon mild kidney injury, serum CYS-C begins to increase 24–48 h before serum Cr increases, and serum CYS-C gradually increases under disease progression [31]. Previous studies showed that serum CYS-C was an independent and significant indicator for predicting AKI secondary to decompensated cirrhosis [32] and after radical gastrectomy [33]. Previous studies have shown that age, gender, and race can affect the level of serum CYS-C in adolescents [34].

Although the CYS-C might be impacted by nonrenal factors such as age, gender, and race, this study found that baseline serum CYS-C concentration was significantly increased in acute pancreatitis patients with AKI compared to that without AKI, suggesting that the increased baseline serum CYS-C might be associated with AKI in patients with acute pancreatitis. Furthermore, baseline serum CYS-C is an independent and significant indicator for AKI in patients with acute pancreatitis according to multivariate logistic regression analysis. AKI in patients with acute pancreatitis could be identified with a sensitivity of 88.9% at specificity of 100% (AUC = 0.948, 95% CI 0.879–1.000) by serum baseline CYS-C (cut-off value = 1.865 mg/L) in our study group. Our finding suggested that baseline serum CYS-C can be a biomarker for AKI in patients with acute pancreatitis. Compared with RIFLE and KDIGO guideline, measurement of baseline serum CYS-C may be an early indicator predicting AKI in patients with acute pancreatitis and could be used routinely in clinical laboratories. If our results are confirmed by other groups, this marker can easily be applied to clinical practice for predicting the occurrence of AKI in patients with acute pancreatitis.

However, there were also some limitations for our study. First, all data were obtained from a single hospital. Whether our findings can be extended to the general population remains in doubt. Second, because of the relatively small sample size of AKI patients, statistical significance in our study should be interpreted with caution.

## 5. Conclusions

In conclusion, the increase of baseline serum CYS-C was associated with AKI in patients with acute pancreatitis. Baseline serum CYS-C may be available for predicting the potential risk of AKI in patients with acute pancreatitis.

## Figures and Tables

**Table 1:** The characteristics of subjects (*n* = 237).

Characteristics	Non-AKI group (*n* = 219)	AKI group (*n* = 18)	*P* value
Age (years)	58.65 ± 15.62	60.83 ± 13.68	0.733
Gender (F/M)	104/115	4/14	0.039
Bun (mmol/L)	6.02 ± 2.85	16.41 ± 8.43	<0.001
Cr (*μ*mol/L)	75.57 ± 23.97	358.37 ± 266.33	<0.001
UA (*μ*mol/L)	292.03 ± 102.65	383.94 ± 171.58	0.025
CYS-C (mg/L)	1.01 ± 0.26	3.64 ± 2.17	<0.001
FBG (mmol/L)	7.70 ± 3.22	7.23 ± 3.86	0.422
AMY (U/L)	852.77 ± 507.00	869.33 ± 872.52	0.220
LIP (U/L)	803.77 ± 518.69	719.80 ± 947.56	0.037
CHE (U/L)	6675.78 ± 1921.20	4685.22 ± 1874.63	<0.001
TP (g/L)	63.47 ± 6.29	58.52 ± 9.52	0.082
ALB (g/L)	37.08 ± 4.64	33.63 ± 6.68	0.057
GLOB (g/L)	26.66 ± 5.28	24.88 ± 6.23	0.286
TBIL (*μ*mol/L)	40.65 ± 46.44	31.34 ± 26.51	0.473
DBIL (*μ*mol/L)	19.69 ± 28.84	12.17 ± 14.99	0.225
TBA (*μ*mol/L)	34.45 ± 69.10	13.15 ± 22.82	0.226
ALT (U/L)	168.13 ± 188.50	172.39 ± 226.09	0.876
AST (U/L)	147.65 ± 209.97	110.83 ± 140.16	0.432
GGT (U/L)	271.03 ± 316.83	270.00 ± 251.27	0.722
ALP (U/L)	130.31 ± 99.02	134.33 ± 93.97	0.560

**Table 2:** Indicators for AKI in patient with acute pancreatitis by univariate logistic regression analysis.

Characteristics	Odds ratio	95% confidence interval	SE	*P* value
Age (years)	1.010	0.977 to 1.043	0.017	0.564
Gender (F/M)	0.316	0.101 to 0.990	0.583	0.048
Bun	1.449	1.244 to 1.688	0.078	<0.001
Cr	1.044	1.026 to 1.062	0.009	<0.001
UA	1.006	1.002 to 1.010	0.002	0.002
CYS-C	284.266	19.459 to 4152.766	1.368	<0.001
FBG	0.953	0.809 to 1.122	0.084	0.561
AMY	1.000	0.999 to 1.001	0.000	0.900
LIP	1.000	0.999 to 1.001	0.000	0.540
CHE	0.999	0.999 to 1.000	0.000	<0.001
TP	0.886	0.819 to 0.959	0.040	0.003
ALB	0.865	0.782 to 0.957	0.052	0.005
GLOB	0.931	0.840 to 1.032	0.053	0.174
TBIL	0.994	0.978 to 1.009	0.008	0.407
DBIL	0.984	0.955 to 1.014	0.015	0.285
TBA	0.991	0.976 to 1.006	0.008	0.233
ALT	1.000	0.998 to 1.003	0.001	0.927
AST	0.999	0.996 to 1.002	0.002	0.470
GGT	1.000	0.998 to 1.002	0.001	0.989
ALP	1.000	0.996 to 1.005	0.002	0.867

**Table 3:** Indicators for AKI in patient with acute pancreatitis by multiple logistic regression analysis.

Characteristics	Odds ratio	95% confidence interval	SE	*P* value
Gender (F/M)	1.300	0.140 to 12.090	1.138	0.817
CYS-C	203.594	13.392 to 3095.230	1.389	<0.001
CHE	1.000	0.999 to 1.000	0.000	0.262

**Table 4:** Diagnostic efficiency of serum CYS-C, Bun, Cr, and UA for AKI in patient with acute pancreatitis.

Characteristics	Sensitivity	Specificity	AUC (95% CI)	Cut-off value
CYS-C	88.90%	100%	0.948 (0.879 to 1.000)	1.865
Bun	83.30%	90.0%	0.915 (0.839 to 0.990)	9.300
Cr	83.30%	99.1%	0.936 (0.851 to 1.000)	171.400
UA	55.60%	73.5%	0.659 (0.514 to 0.803)	342.050

## Data Availability

The data supporting the findings of this study were listed in the supplementary file (available here).

## Supplementary Materials

Supplementary Materials The data (Data Availability.xlsx) supporting the findings of this study were listed in the supplementary materials.
